# Supplementary material for: Experimental evaluation of 3D printed spiral phase plates for enabling an orbital angular momentum multiplexed radio system
Source: R Soc Open Sci. 2019 Dec 11;6(12):191419. doi: 10.1098/rsos.191419 (PMC6936294; doi:10.1098/rsos.191419)
Supplement: Supplementary Material [file rsos191419supp1.docx]

**Supplementary Material**

3D complex radiation patterns for various combinations of horn / SPP / lens.

| **Magnitude (dBi)** | **Phase (deg)** |
| --- | --- |
| 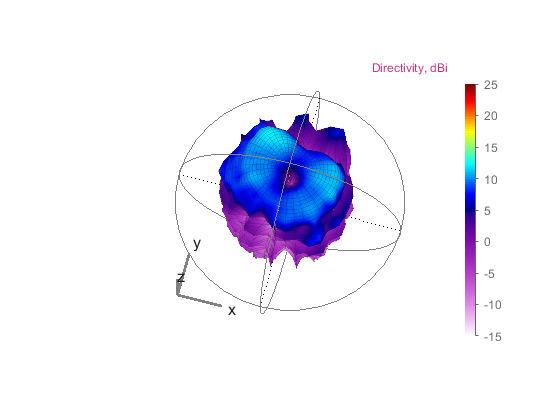  SPP1DirectivityPattern | 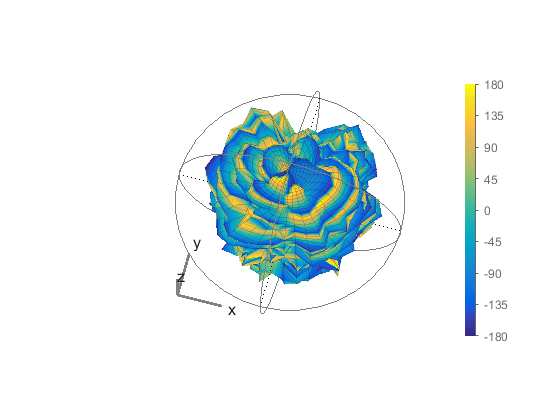  SPP1PhasePattern |
| 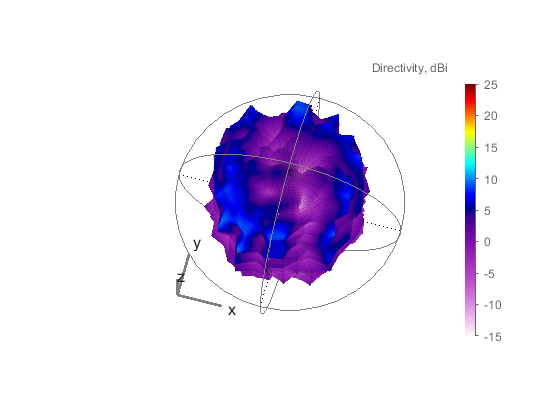  SPP2DirectivityPattern | 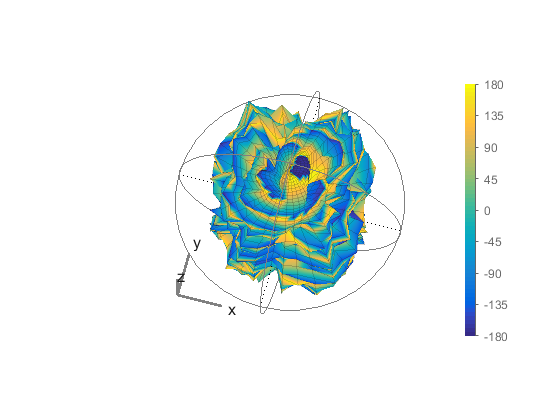  SPP2PhasePattern |
| 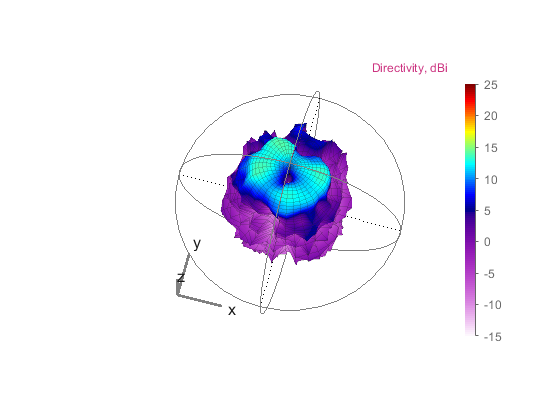  SPP1Lens11DirectivityPattern | 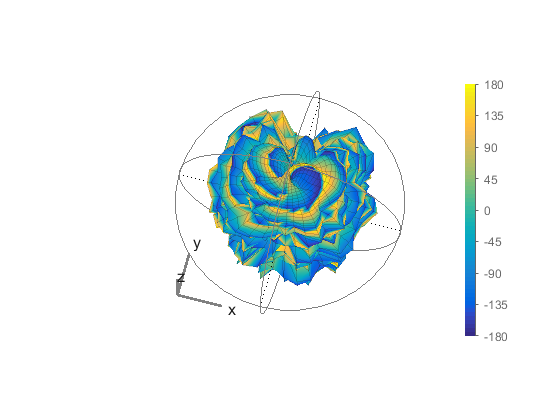  SPP1Lens11PhasePattern |
| 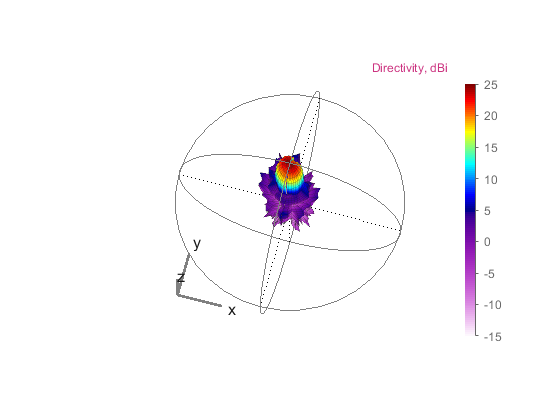  HornLens13DirectivityPattern | 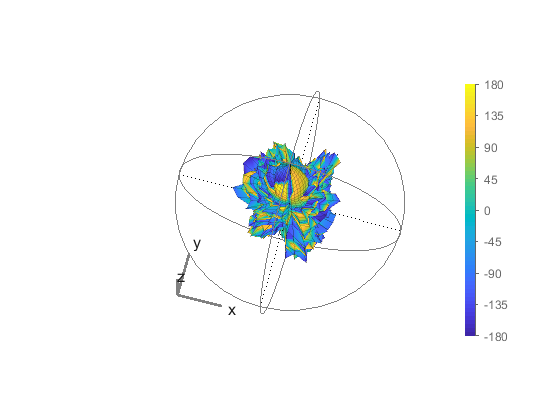  HornLens13PhasePattern |
| 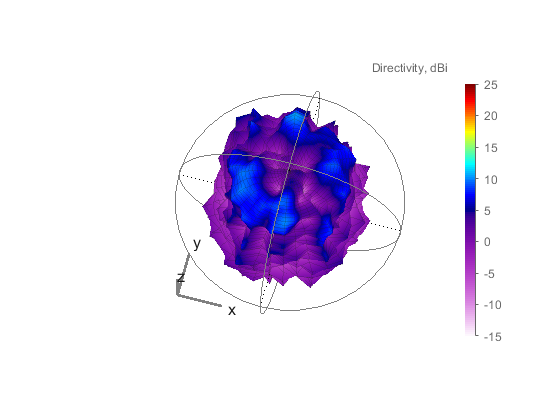  SPP2Lens11DirectivityPattern | 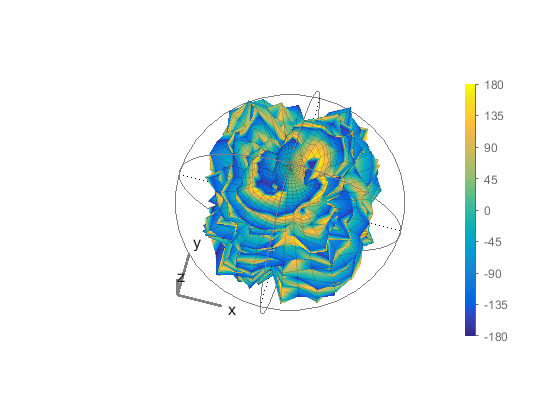  SPP2Lens11PhasePattern |
| 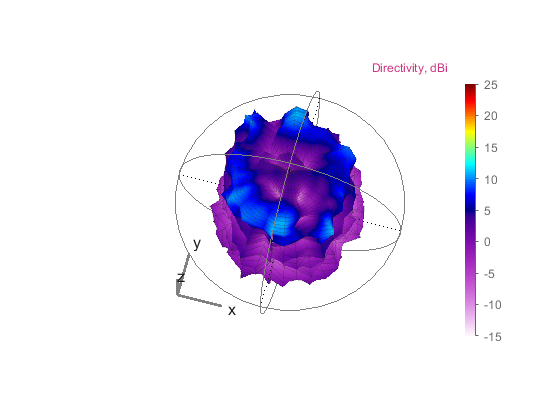  SPP2Lens13DirectivityPattern | 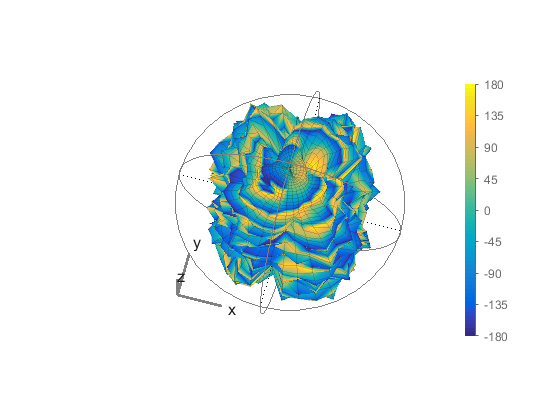  SPP2Lens13PhasePattern |
